# Uncommon Presentation of a Schwannoma in the Radial Nerve: A Surgical Case Report

**DOI:** 10.7759/cureus.80865

**Published:** 2025-03-20

**Authors:** Felix Rivera Troia, Carlos J Perez Lopez

**Affiliations:** 1 Orthopedic Surgery, Ponce Health Sciences University, Ponce, PRI

**Keywords:** neuronal tumors, orthopedic surgeries, peripheral schwannoma, radial nerve, rare tumors

## Abstract

Schwannomas are uncommon, mostly benign nerve sheath tumors that typically present as painless or intermittently painful lumps. They are most commonly found on the flexor surfaces of the body, with occurrence in the radial nerve being exceptionally rare. We present the case of a 55-year-old male with a five-month history of a progressively enlarging, mobile mass on the posterior aspect of the right arm. The patient presented with a transient, “shock-like pain” that failed to respond to conservative management. Imaging confirmed a peripheral nerve schwannoma located deep in the triceps posterior fascia along the lateral head of the triceps within the radial nerve. Due to persistent symptoms and failure of conservative treatment, surgical excision of the neural tumor was recommended. This case highlights the clinical presentation and surgical management of a rare radial nerve schwannoma.

## Introduction

Peripheral nerve schwannomas are benign, slow-growing tumors arising from neuronal tissue and comprise less than 10% of all soft tissue tumors [1,2]. They originate from Schwann cells, which produce the myelin sheath around the peripheral nerves. These lesions are typically encapsulated and composed of three distinct layers: a nerve layer, a fibrous layer, and a transitional layer [3]. Studies suggest these tumors have no significant sex predilection, and while there is no strict age range for presentation, they most commonly appear around the fourth decade of life [4]. Although rare, when they do occur, schwannomas tend to arise on the flexor surfaces of the body [5]. Clinically, schwannomas often present as painless, slow-growing, mobile masses. However, depending on their size and location, they can cause neurological symptoms, such as pain, paresthesia, or motor deficits, due to nerve compression or irritation [6-8].

Masses in the upper extremity are more frequently found in the hand, particularly in the fingers, rather than proximally [9]. The majority of these lesions are benign, most commonly ganglion cysts or giant cell tumors [9,10]. However, peripheral nerve tumors, although less common, are significant lesions that should be considered in the differential diagnosis of upper extremity masses. Recognizing schwannomas is particularly important, as their presentation can closely mimic more common lesions, potentially leading to misdiagnosis and delaying appropriate management [11]. The radial nerve is a particularly infrequent location for this neural tumor, with only a few cases documented in the literature [2,7,12].

This case highlights the clinical presentation and surgical management of a peripheral nerve schwannoma in an uncommon location, the radial nerve. Furthermore, it highlights the importance of including schwannomas in the differential diagnosis of upper extremity masses.

## Case presentation

A 55-year-old male presented to the clinic with a five-month history of a gradually enlarging mass on the posterior aspect of his right arm. On inspection, no visible mass was apparent; however, palpation revealed a mobile lesion located deep in the triceps muscle in the distal third of the posterior aspect of the arm. The mass was tender only on palpation, with the patient describing the pain as a transient, “shock-like sensation” radiating down the arm to the fingers, rated as 8/10 on the pain scale.

**Figure 1:**
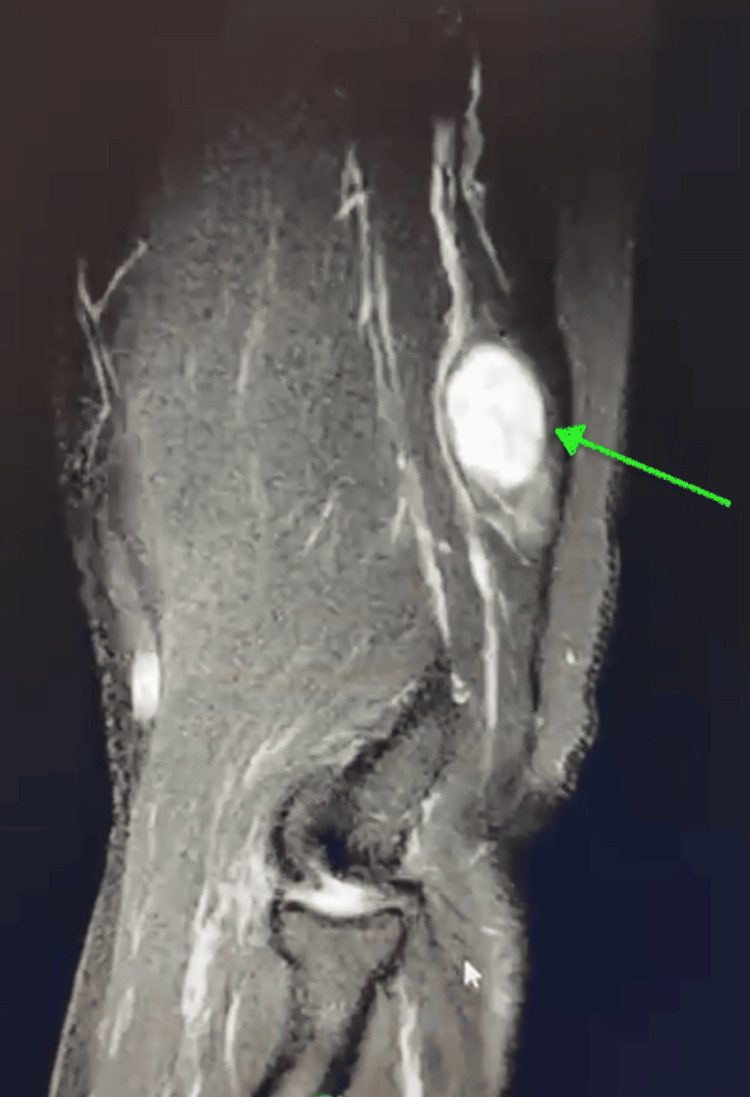
Sagittal view of a contrast-enhanced MRI of the right arm demonstrating a peripheral nerve tumor (green arrow) in the radial nerve

**Figure 2:**
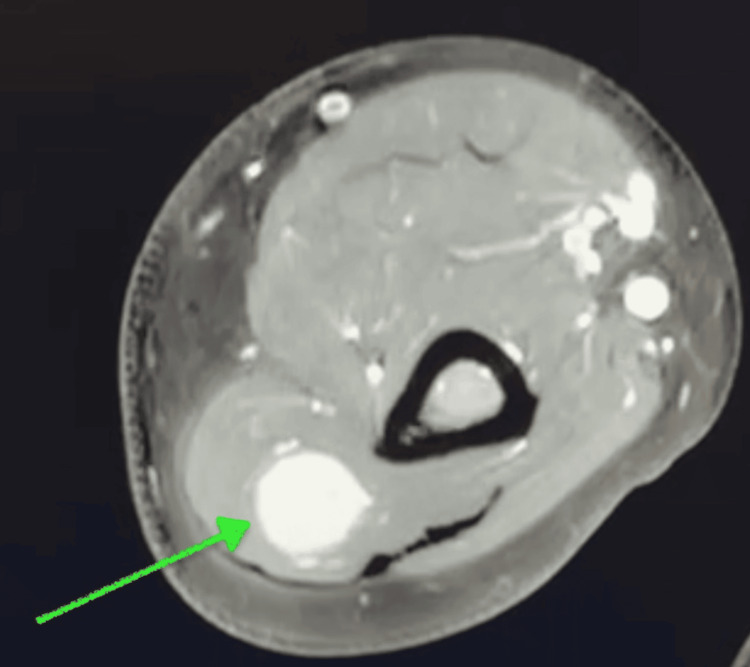
Axial view of a contrast-enhanced MRI of the right arm showing a peripheral nerve tumor (green arrow) deep to the triceps posterior fascia along the lateral head of the triceps

On physical examination, the patient demonstrated full range of motion at the shoulder, elbow, and wrist joints, with normal wrist extension. No associated muscle weakness or sensory deficits were noted. He described himself as physically active, regularly lifting weights, and had attempted to manage his symptoms with hot and cold patches, although these failed to provide adequate relief. Given the location and presenting symptoms, a contrast-enhanced MRI was ordered to aid in the diagnosis. Imaging revealed a well-circumscribed fusiform homogeneously avidly enhancing mass within the radial nerve deep to the triceps posterior fascia along the lateral head of the triceps (Figure 1, Figure 2). Given he had failed conservative management, surgical excision of the mass was recommended.

Surgical procedure

**Figure 3:**
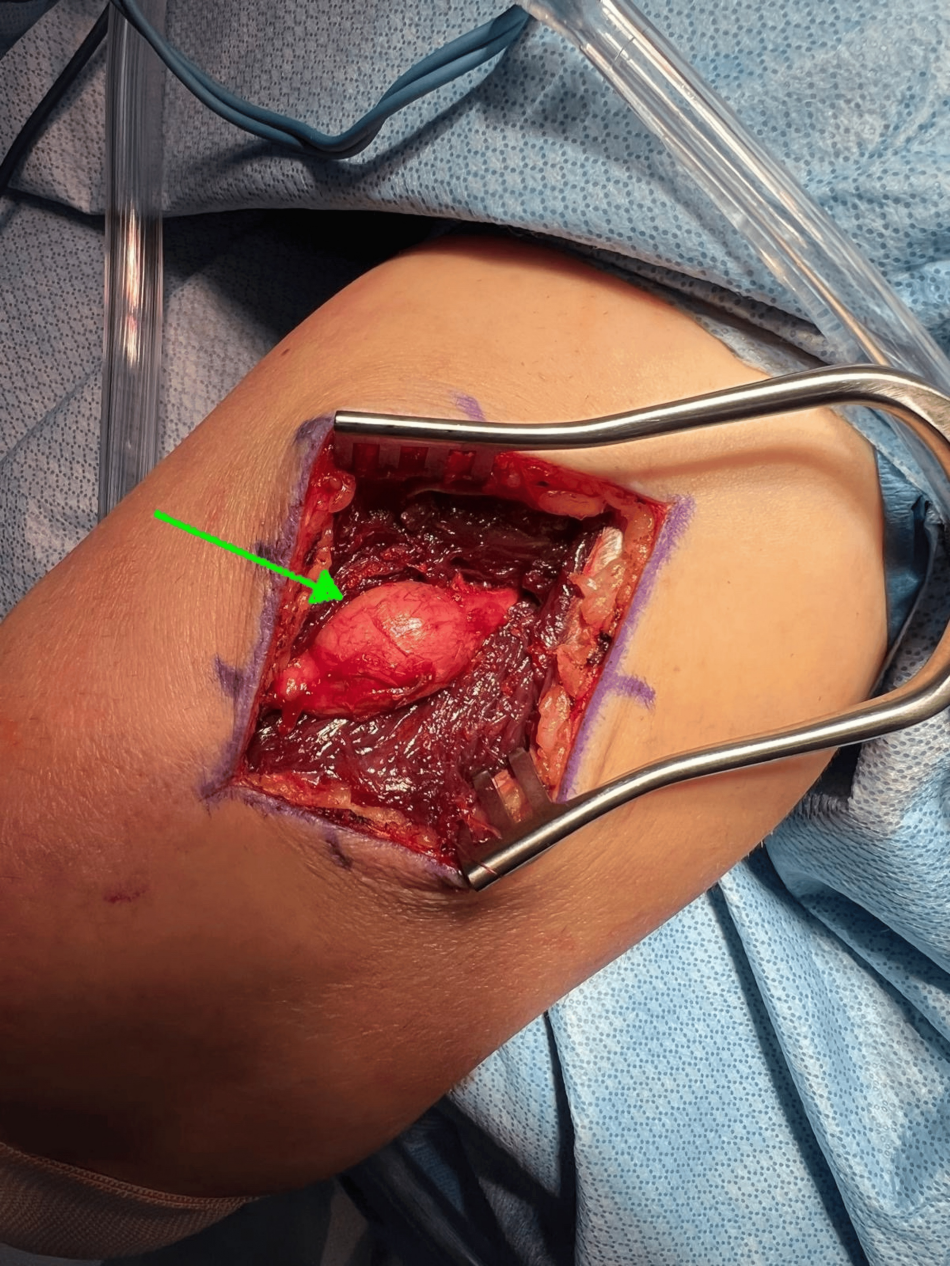
Interval between triceps muscles demonstrating the peripheral nerve tumor within the radial nerve sheath (green arrow)

**Figure 4:**
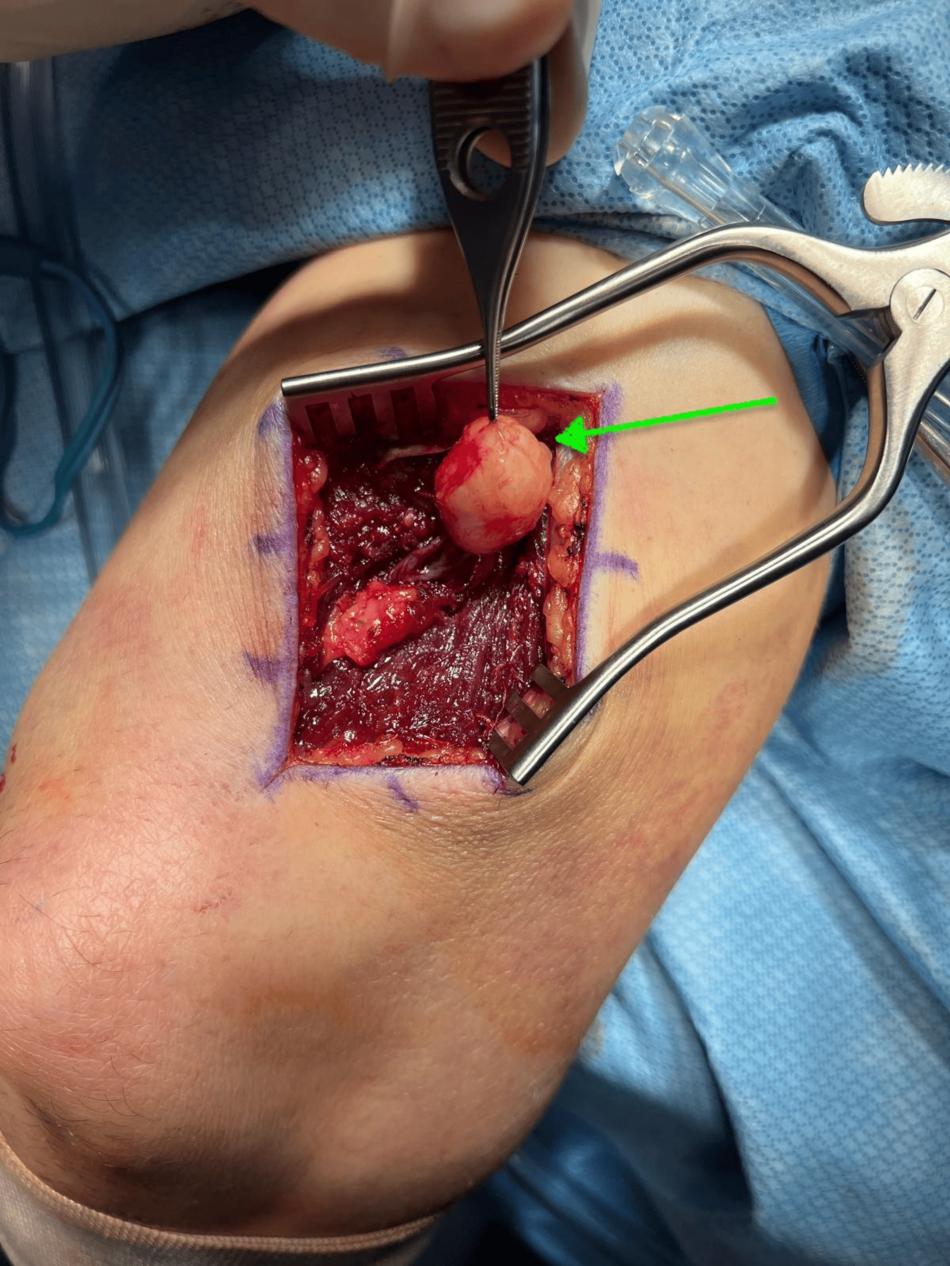
Peripheral nerve tumor exposed (green arrow)

**Figure 5:**
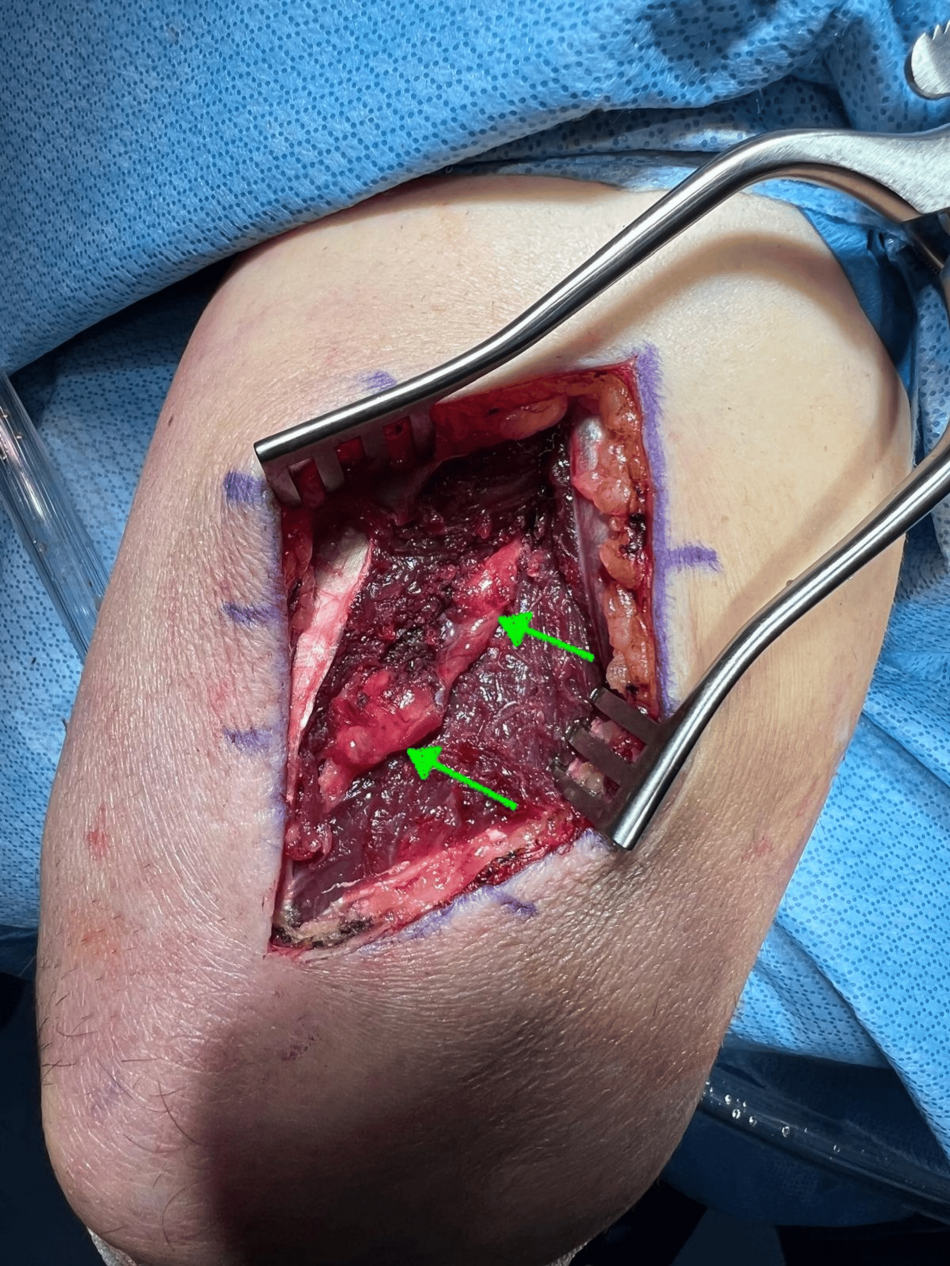
Radial nerve after excision of the peripheral nerve mass (green arrows)

The patient was placed in the lateral decubitus position, with all extremities well padded. The right upper extremity was prepped and draped in the usual sterile fashion with the arm placed on a well-padded arm holder. A posterior incision was made over the distal arm, allowing exploration deep into the triceps muscle to expose the radial nerve (Figure 3). Under microscopic guidance, the radial nerve was carefully dissected. Bipolar cautery and microvascular instrumentation were used to carefully expose the nerve sheath. The sheath was then incised under direct visualization, and meticulous dissection was performed to separate it from the tumor (Figure 4). The tumor was carefully isolated, mobilized away from the radial nerve, and excised (Figure 5). Following tumor removal, the nerve sheath was approximated using microvascular sutures. The area was copiously irrigated, the triceps fascia closed, and the wound was closed in layers.

The pathology report confirmed the diagnosis of a peripheral nerve schwannoma. Postoperatively, the patient was encouraged to begin early range of motion exercises as tolerated and was scheduled to return to the clinic at one week, three weeks, and six weeks for follow-up. At five-year follow-up, the patient remained asymptomatic, with no signs of recurrence. Physical examination was unremarkable, with full range of motion at the shoulder, elbow, and wrist joints. The patient reported being satisfied with the outcome of the procedure and continues to exercise with no functional limitations.

## Discussion

Upper extremity masses are a common clinical finding in orthopedic and general surgery practices, with benign lesions such as giant cell tumors, lipomas, and ganglion cysts among the most prevalent [10]. Schwannomas, although mostly benign, are much less common in this region, particularly in the radial nerve, with only a few cases reported in the literature [2,7,12]. These lesions can cause neurological symptoms, such as paresthesia or motor deficits, due to adjacent nerve compression or irritation [7,8].

MRI is the preferred diagnostic tool for detecting peripheral nerve schwannomas due to its noninvasiveness and high resolution for soft tissue structures [13]. Some studies have assessed its diagnostic accuracy and suggest that it offers sufficient detail to effectively distinguish between different types of schwannomas [14]. Management of these tumors depends on the severity of symptoms. However, some authors recommend early surgical excision in cases of severe symptoms, noting positive outcomes associated with this approach [15,16]. Bhat et al. reported two cases of ancient peripheral nerve schwannomas, one of which involved the radial nerve in a patient of similar age with physical exam findings comparable to those in our case [17]. Similarly, Hamdaoui et al. described a younger patient with a mass in the left arm who experienced similar shock-like sensations along the radial nerve distribution and was ultimately diagnosed with a radial nerve schwannoma [6]. However, unlike our case, Hamdaoui et al. used ultrasound to aid in their diagnosis. Nevertheless, in both cases, the patients underwent successful surgical excision of the neural tumor without recurrences, consistent with the approach and outcomes observed in our study.

Early surgical excision is considered beneficial for symptomatic schwannomas, providing favorable outcomes and minimizing the risk of recurrence, consistent with previous reports and the findings of this case.

## Conclusions

This case highlights the presentation and surgical management of a peripheral nerve schwannoma in the radial nerve, an uncommon location for such tumors. Additionally, it highlights the importance of including schwannomas in the differential diagnosis of upper extremity masses. MRI is suggested to be a valuable tool in preoperative planning. Furthermore, surgical excision proved advantageous when performed safely with meticulous nerve-sparing techniques, as evidenced by the satisfactory functional outcome reported by the patient.

## References

[1] Majumder A, Ahuja A, Chauhan DS, Paliwal P, Bhardwaj M (2021). A clinicopathological study of peripheral schwannomas. Med Pharm Rep.

[2] Phan D, Ton NT, Bui TH, Tran HN, Truong VT (2023). Ancient schwannoma of the radial nerve: a case report. Cureus.

[3] Ying GY, Yao Y, Shen F, Wu ZY, Chen CM, Zhu YJ (2018). Percutaneous endoscopic removal of cervical foraminal schwannoma via interlaminar approach: a case report. Oper Neurosurg (Hagerstown).

[4] Albert P, Patel J, Badawy K, Weissinger W, Brenner M, Bourhill I, Parnell J (2017). Peripheral nerve schwannoma: a review of varying clinical presentations and imaging findings. J Foot Ankle Surg.

[5] Senol N, Yilmaz O (2015). A rare type of peripheral nerve sheath tumor: radial nerve schwannoma. Turk Neurosurg.

[6] Hamdaoui J, Elkamch H, Gharib N, El Mazouz S, Abbassi A, Hafidi J (2022). Schwannoma of the radial nerve: a case report. Pan Afr Med J.

[7] Lui TH, Li CH (2020). Nerve-preserving endoscopically assisted resection of schwannoma of the radial nerve. Arthrosc Tech.

[8] Severo AL, Alencar Neto DM, Lemos MB, Duarte MP, Tagliari I (2024). Posterior interosseous nerve syndrome due to schwannoma - a case report. Rev Bras Ortop (Sao Paulo).

[9] Zyluk A, Mazur A (2015). Statistical and histological analysis of tumors of the upper extremity. Obere Extrem.

[10] Fang L, Abdullah S, Tadrousse K (2025). Outcome predictors of clinically diagnosed benign upper extremity soft tissue masses: a retrospective study. J Orthop Rep.

[11] Magalhães M, Pereira D, Oliva H (2019). Peripheral nerve schwannomas: a literature review. Arq Bras Neurocir.

[12] Wong YC, Ho PC, Tse WL, Chow LT, Wong CW (2014). Malignant schwannoma of the radial nerve with unusual presentation: a case report. Hand Surg.

[13] Zhai H, Lv Y, Kong X, Liu X, Liu D (2019). Magnetic resonance neurography appearance and diagnostic evaluation of peripheral nerve sheath tumors. Sci Rep.

[14] Isobe K, Shimizu T, Akahane T, Kato H (2004). Imaging of ancient schwannoma. AJR Am J Roentgenol.

[15] Barber CM, Fahrenkopf MP, Adams NS, Naum SC (2018). Multiple peripheral schwannomas. Eplasty.

[16] Zipfel J, Al-Hariri M, Gugel I (2021). Surgical management of sporadic peripheral nerve schwannomas in adults: indications and outcome in a single center cohort. Cancers (Basel).

[17] Bhat AK, Acharya AM, Narayanakurup JK, Shankar V (2017). Ancient schwannoma of radial nerve: a report of two cases. J Hand Surg Asian Pac Vol.

